# Design of Early Validation Trials of Biomarkers

**Published:** 2007-02-25

**Authors:** Daniel Normolle, Mack T. Ruffin, Dean Brenner

**Affiliations:** a Department of Radiation Oncology, University of Michigan Medical School, University of Michigan Comprehensive Cancer Center Biostatistics Unit; b Department of Family Medicine, University of Michigan Medical School; c Department of Internal Medicine and Department of Pharmacology, University of Michigan Medical School, and VA

**Keywords:** mass screening, research design, decision theory

## Abstract

The design of early-phase studies of putative screening markers in clinical populations is discussed. Biological, epidemiological, statistical and computational issues all affect the design of early-phase studies of these markers, but there are frequently little or no data in hand to facilitate the design. Early-phase studies must be designed as part of a development program, considering the final use of the marker, directly informing the decision to made at the study’s conclusion. Therefore, they should test for sensitivity and specificity that would be minimally acceptable to proceed to the next stage of development. Designing such trials requires explicit assumptions about prevalence and false positive and negative costs in the ultimate target population. Early discussion of these issues strengthens the development process, since enthusiasm for developing technologies is balanced by realism about the requirements of a valid population screen. Receiver operating characteristic (ROC) curves, which are useful descriptive tools, may be misleading when evaluating tests in low-prevalence populations, because they emphasize the relationship between specificity and sensitivity in the range of specificity likely to be too low to be useful in mass screening applications.

## Introduction

Prototypical research programs for early detection markers, using technologies such as SELDI, have been presented by Pepe (Pepe 2001). This five-phase concept leaves many degrees of freedom for the design of studies within the phases. As investigators in an Early Detection Research Network Clinical and Epidemiological Center (EDRN-CEC), we are interested in the design of trials which fit into the early part of Pepe’s Phase II, which could be called early validation or pre-validation studies. These would typically be the first studies conducted in association with investigators independent of the laboratory that developed the assay. They may be on archived samples, or samples that are collected prospectively. Since both biological samples that are prospectively obtained and archived samples that have been drawn from clinical protocols are expensive to harvest, document and maintain, pre-validation tests of assays must be carefully designed for the samples’ efficient use.

The context of this discussion may be made more concrete by consideration of a simple example. Suppose a developmental laboratory has assayed twenty serum samples that have been collected over time from colon cancer patients, and twenty serum samples from patients who had colonoscopies, but did not have cancer. The assay produces a single numerical value, and the laboratory reports a two-standard deviation difference between the cases and the controls, generating significant interest. What is the next step?

In this paper, we discuss criteria for design of these trials, and contend that, rather than consider the trial as a standalone event, it must be designed in the context of the research program, considering the ultimate application of the marker. Critical elements are the minimum criteria for acceptable sensitivity and specificity, the method of the estimation of the endpoints, and the nature of the cases and controls to be tested. We will demonstrate these issues primarily by means of small Monte Carlo simulation experiments that are representative of those used to justify clinical trial designs.

## General design criteria

The key features of early validation trials are that they are part of a development program and they are expensive. The implication of the first feature is that the least expensive internally valid trial may not be the best trial to conduct, since it may not contribute to the developmental decisions that must be made. The second feature is generally realized as a severe constraint on the number of samples available.

The intended application of the marker determines the structure of the developmental program, but a major goal of the research program is to define the application. The research program therefore has a number of sequential, conditional decisions. Trials should produce sufficient information so that these decisions, which may include termination of the development program, can be made with confidence. This implies that trials should be adequately powered to test hypotheses. The following questions, each of which could be reformulated as a testable hypothesis, are commonly unanswered in the early phase of a developmental program and would be good candidates for goals of early validation trials:

### Clinical vs. population screen

Will the marker be used as a screen of a large, untested population, where prevalence is likely to be low, or in a clinical setting, where the prevalence is likely to be orders of magnitude higher, but patients are likely to have other disease?

### Standalone vs. panel

Is the marker to be used in isolation, or in combination with existing markers, or markers yet to be tested? Will it be used in combination with demographic and risk information?

### Asymptomatic vs symptomatic normals

Will it be used to distinguish patients with cancer from other patients, or patients with pre-cancerous conditions, or subjects with biological risk factors for cancer, from healthy people?

In the colon cancer example, the next developmental step may be to assess the adequacy of the sensitivity and specificity of the marker in patients with adenomas or cancers versus healthy volunteers who are verified cancer-free. A design that simply estimates the sensitivity and specificity may not be sufficient, since it may present a result that is scientifically valid, but insufficiently conclusive to determine the course of the research program. For instance, consider a trial that recruits twenty cases and twenty controls (based on 80% power for a two-sample t-test on a continuous marker having a two-standard-deviation effect size) and has the result in Table 1.

The numbers in parentheses are the 95% likelihood ratio confidence intervals (Collett 1991) for the specificity (assessed in the controls) and the sensitivity (assessed in the cases). The intervals cover too large a range to determine if the marker is adequate, and the study will have to be repeated with a larger sample size. This could have been avoided by considering the minimal criteria for developing the marker as a population screen at the design phase of the study.

## Example of a design for a pivotal trial

The answer to the first question in Section 2 hinges upon the sensitivity and specificity of the test. Constrained by a limited number of samples, it is tempting to design an early validation trial by asking the question, “How sensitive and specific is the marker?” This leads to a trial with an arbitrary sample size and an equivocal result, since it will be driven by the width of the estimated confidence intervals, or, more likely, the width of the estimated confidence intervals will be determined by an the size of an easily obtained sample. A better question is, “Are the sensitivity and specificity big enough to develop this test as a population screen?”

The big-enough criterion is defined by decision theory. Consider the risk-minimizing decision rule in Equation 1 (for a derivation, see Hand 1981).

(1) $$\begin{array}{l} \begin{array}{cc} \text{If }\frac{\hat{P}(x|+)}{\hat{p}(x|-)}>\frac{\left(1-\pi \right)}{\pi }\cdot \frac{C_{+}}{C_{-}}\;\text{then} & \text{Classify }x\in +\\ \text{otherwise} & \text{Classify }x\in - \end{array}\;\;\; \end{array}$$

This decision rule applies to all statistical classifiers, including Fisher’s linear (or quadratic) discriminant analysis, nearest-neighbor classification, logistic regression, recursive partitioning trees (also known as CART), neural networks and vector support machines. Different classifiers are simply different estimators for the ratio of class-conditional probability density functions on the left-hand side of the decision rule. The right-hand side of the decision rule represents the level of evidence required to classify a patient represented by a vector of variables, *x*, as a case. The value *π* is the prevalence of cases in the target population, which cannot in general be estimated from a case-control study. C_−_/C_+_ is the estimated ratio of the cost of a false negative to a false positive error. The prevalence and cost ratios are inherent in any statistical classification problem; if they are not considered, statistical packages set the right-hand side of Equation 1 equal to 1, which may not be a sensible value. The risk-minimizing decision rule is directly related to the Receiver Operating Characteristic (ROC, Swets, et al. 2000) curve by Equation 2:

(2) $$\text{RO}{\text{C}}^{\prime }=\frac{\left(1-\pi \right)}{\pi }\cdot \frac{C_{+}}{C_{-}}$$

**Figure 1:**
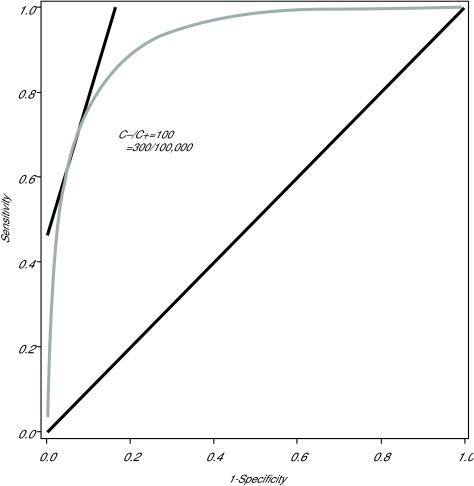
ROC curve for a normally-distributed univariate random variable. The sensitivity and specificity values that minimize the expected classification cost for the given prevalence and C_−_/C_+_ values is displayed.

That is, given an ROC curve, the optimal combination of sensitivity and specificity are determined by the point on the curve with the slope as described in Equation 2. The application of the optimal rule to the preliminary data generated by the colon-cancer screening example, assuming a prevalence of 300/100,000 and a C_−_/C_+_ ratio of 100, is demonstrated in Figure 1.

This suggests a trial design algorithm for the question “Are the sensitivity and specificity of the marker large enough to warrant its use as a population screen?”

1. Determine a consensus on: the prevalence in the screening population, π; the cost ratio, C_−_/C_+_; the sensitivity, γ_1_, required for the marker as a population screen.
2. Plug these assumptions into Equation 1 to determine the required specificity, δ_1_.
3. Select the significance level, α, and power, 1-β. The significance level could be adjusted to maintain the experiment-wise Type I error. Since this is an early-phase test, α can be set relatively high (say, 0.1).
4. Define the *null* hypotheses γ ≤ γ_0_ < γ_1_ and δ ≤ δ_0_ < δ_1_, that the sensitivity and specificity are not large enough, on the cases and controls, respectively.
5. Determine the number of cases and controls to achieve the stated power for one-sided exact binomial tests and the stated significance levels if γ = γ_1_ *and* δ = δ_1_. It is likely that the number of controls will be larger than the number of cases because the required specificity is likely to be higher than the required sensitivity.

## Estimation of sensitivity and specificity

In the previous section, we discussed the criteria for the null hypothesis of a clinical trial to estimate sensitivity and specificity. In a randomized trial to test the difference between the means of two populations, the criteria described, along with a significance level (α) and power (1-β) are sufficient to determine the sample size. But the experimental situation in a trial of a prospective marker is usually more complicated, because part of the study is the construction of a statistical classification model, comprising both the selection of prediction variables from a set of candidate markers (which, for example, in a SELDI model, may be the set of all identified peaks in the spectrum, or even all the M/Z values in the spectrum, and hence very large in number) and the construction of the classification function. The selection of variables and construction of the classifier is generally referred to as training, and the estimation of sensitivity and specificity is termed testing. Some authors (e.g., Burke4), further distinguish between a testing set and a validation set.

The sensitivity, specificity and other measures of the quality of a statistical classifier are characterized by using the classifier to evaluate samples of known class membership (case or control) and determine the proportions of cases and controls correctly classified. Using all of the available cases and controls to train the classifier, and then submitting all of those cases to the classifier, called re-substitution, tends to produce a phenomenon known as over-fitting, which causes the estimates of sensitivity and specificity to be optimistically biased. Alternatives to re-substitution are the use of an independent test set, which is not used to train the classifier function, and cross-validation. In cross-validation, observations are held out one at a time, and the classifier is trained without considering the withheld observation. Then, the withheld observation is classified, thereby achieving independence of training and testing at the cost of a small reduction in the efficiency of the training. Cross-validation is easy and inexpensive when used with discriminant analysis, logistic regression or nearest-neighbor classification, but is more difficult or, at least, computationally expensive, with other classification methods.

**Figure 2:**
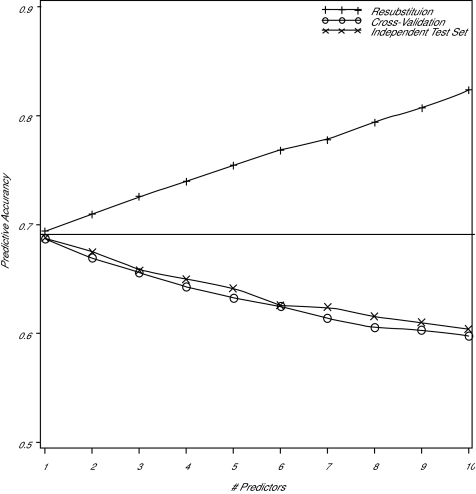
Monte Carlo simulation (10,000 replications) demonstrating predictive accuracy of a statistical classifier based on 15 training cases and 15 training controls. Re-substitution estimates accuracy by reclassifying the training observations using classifying function, cross-validation recalculates the classifier by leaving the training observation to be classified out of the parameter estimation, and an independent test set is used for the third estimator. The horizontal line indicates the optimal accuracy that can be achieved if the within-class distributional parameters are known rather than estimated from the training set.

The effect of re-substitution is demonstrated by the display of the Monte Carlo experiment in Figure 2. In this experiment, the number of training observations for Fisher’s linear discriminant function (LDF) was held constant at 30, half cases and half controls. The number of variables used to discriminate between the two classes, graphed on the horizontal axis, was varied from 1 to 10, but the total separation between the classes was held constant; the experiment was replicated a total of 10,000 times. The profiles display the mean, over 1,000 replications, of the accuracy, equal to the fraction of all observations (cases and controls) correctly classified, using the three estimation methods. The horizontal line shows the accuracy possible if the class-conditional probability distribution functions, and hence the coefficients of the LDF, were known rather than estimated from the training sample. It is seen that, as the number of variables used to classify the observations is increased, the positive bias of the re-substitution estimate of the accuracy significantly increases. Also, the cross-validation estimator of accuracy is close to, but slightly less than, the independent test set estimator. The accuracy of the latter two estimators declines as the number of variables increases because the number of coefficients that must be estimated (in the LDF, *2p* +*p(p*+*1)/2*) is increasing. The independent test set estimator appears to be slightly more accurate than cross-validation estimator because one more training observation is available for coefficient estimation, but this slight increase in accuracy is purchased at the cost of the test set.

**Figure 3:**
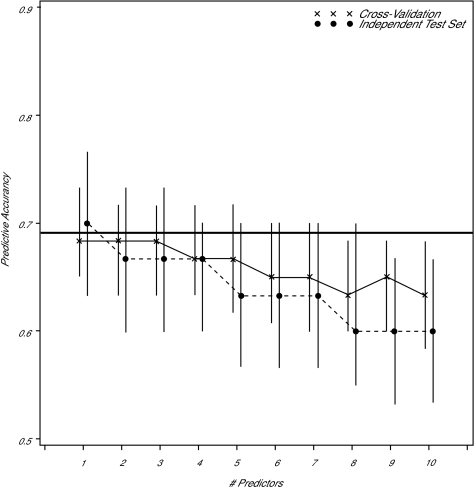
Monte Carlo simulation (10,000 replications) demonstrating predictive accuracy of a statistical classifier based on 30 cases and 30 controls. Cross-validation estimator pools all 60 cases and recalculates the classifier by leaving out the training observation to be classified. The independent test set classifier splits the cases and controls in half and uses the first half for training and the second half for testing. The vertical bars connect the first and third quartiles of the observed accuracies, while the profiles connect the medians. The horizontal line is defined as in Figure 2.

From the experiment described in Figure 2, we observe that, even when the variables to be used in the classifier are fixed in advance, the re-substitution estimator significantly over-estimates the accuracy, the over-estimation increases as the number of parameters estimated, relative to the number of training samples, increases, and the cross-validation estimator achieves nearly the same accuracy as the independent test set estimator. Given a fixed number of cases and controls, should an independent test set be held out, or should the classifier be cross-validated? Figure 3 demonstrates an answer to the question when the classifier is based on a linear function of the data (e.g. LDF or logistic regression) and the variables to be used in classification are known in advance. Here, sixty observations are classified two ways, by dividing them into equal-sized training and testing sets, and by using all sixty observations in a cross-validated classifier. It is seen that, since there are almost twice as many training observations available to the cross-validated classifier than the independent test set classifier, it is more accurate, and displays less variation in accuracy. This example demonstrates that, if the variables used for prediction are known, cross-validation, if possible (given the method of classification), uses the observations more efficiently than holding some aside from coefficient estimation as a test set.

The situation is complicated when the selection of variables is part of the training. As is pointed out elsewhere in this volume (Burke 2004), estimates of sensitivity, specificity and accuracy based on an independent test set are no longer unbiased when the test set has been used repeatedly as part of an iterative variable selection process. In this case, the testing set rather than the training set will be over-fit, and sensitivity, specificity and accuracy will still be over-optimistically estimated. As we have seen, where computationally feasible, the cross-validation estimator makes more efficient use of the data than a separate testing sample. Hence, at the variable-selection stage, there is no justification for using a holdout sample if cross-validation is possible, but the final estimates of sensitivity, specificity and accuracy thereby derived are not of the same quality as in the case where the variable set is fixed, and a separate validation set must be used after the variable selection process to ensure unbiased estimates of sensitivity and specificity.

## Size of the training set

In Section 3, we proposed a design to test a hypothesis set appropriate for a pre-validation trial. The sample sizes derived there apply to the test or validation set, given that the variables have been selected and the classifier has been trained. How large should the training set be? There is no answer to this question general enough to apply to all statistical classifiers, and designers of trials frequently resort to heuristics, such as ‘10 observations per variable.’ In order to give some structure to the problem, we propose three sets of experimental situations: the variables have been selected and only parameter estimation is necessary; variables must be selected among thousands of candidates, as in SELDI or microarray analysis; variables must be selected from a limited number.

*Parameter estimation only:* when the variables have been selected, then unbiased estimates of sensitivity and specificity are available through cross-validation, so that the testing set is the training set, and arguments like those in Section 3 may suggest an appropriate sample size. If estimates of within-class distributions of the markers are available, Monte Carlo simulations can be used to test if a given increase in the sample size would significantly increase the precision of the parameter estimates and thereby increase the quality of the estimates of sensitivity and specificity.

*Variables must be selected among thousands of candidates:* the phenomenon of the inflation of the Type I error rate when performing many univariate hypothesis tests has been well-known at least since Fisher’s work (Fisher 1935) in the 1930’s. In this case, p-values corresponding to univariate hypothesis tests may be useful to order the possible indicators with respect to further interest, but they are not directly interpretable without some further adjustment by, for instance, inflating the estimate of standard errors in the denominators of t-tests (Dutoit 2002), using permutation tests to adjust p-values (Efron, et al. 2001), or a minimum absolute effect size criterion to t-statistics. This makes it difficult, if not impossible, to apply the usual power calculations, or even to effectively simulate the process. It is also more likely that a nonlinear classification techniques will be more appropriate for this application. Possibly the only design criteria that can be applied are ‘10 observations per variable’ or, simply ‘as many as possible.’

*Variables must be selected from a limited number:* this represents a situation somewhere between the first case, where simple simulations and, possibly, analytic criteria, can determine a useful sample size, and the second, where even simulated solutions are intractable. Because variable selection methods are sequential, analytic derivations of Type I error rates and power are difficult if not impossible, but the variable selection process can be simulated. A typical situation is:

1. There are a fixed number of samples, say 50 cases and 50 controls.
2. Variable selection will be performed on the training set, and hence,
3. A holdout (validation) sample will be reserved for unbiased estimates of sensitivity, specificity and accuracy.

**Figure 4:**
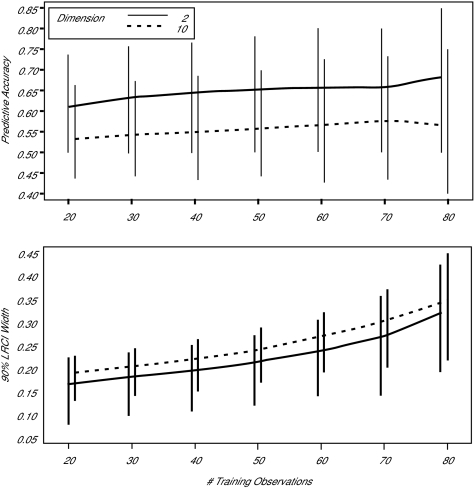
Monte Carlo simulation of 14,000 trials of 100 cases and controls divided into a training set and a validation set. Variables are selected using stepwise linear discrimination, and then used to classify the observations in the validation set. The top panel shows the median predictive accuracy, where the vertical bars indicate the 9^th^ and 95^th^ percentiles. The bottom bars show the median, 5^th^ percentile and 95^th^ percentile of the width of the 95 percent likelihood ratio confidence interval of the predictive accuracy.

How should the samples be allocated between the training and validation sets? Consider the experiment depicted in Figure 4. In this Monte Carlo experiment, replicated 14,000 times, either 2 or 10 discriminator variables were generated from a multi-variate normal distribution with a randomly generated correlation structure and variance adjusted so that the Mahalanobis distance between the two populations was constant across the simulations. Stepwise linear discrminant analysis was used to select variables based on a training set of *n* observations, and then the final discriminator was validated on the remaining 100-*n* cases and controls. The distribution of the achieved accuracy as the training sample size (*n*) was increased from 20 to 80 (and the validation sample size was decreased from 80 to 20) is graphed. If the population parameters were known, predictive accuracy of about 0.68 is possible. In the top frame, when *p*=2, the predictive accuracy increases from 0.61 to 0.66. But, when *p*=10, a more realistic situation, increasing the training sample from 20 to 80 has almost no effect; the sample size is inadequate in any case. The bottom frame shows the width of the likelihood ratio confidence interval around the accuracy, which is a function of the validation sample size. It is seen that, when the number of variables is small, over-allocation of observations to the training set, thereby reducing the size of the test set, produces unnecessarily large confidence intervals. When the number of variables is large, in effect, the training sample is too small in all cases, so that allocation of cases to the training sample only increases the width of the final confidence interval, without any significant gain in accuracy.

## Discussion

At the early validation stage of the research program, there are a number of possible objectives to a trial. Some non-pivotal goals might be to technically develop (tweak) an assay on a broader, more representative or better-described sample, or characterize sources of variation in an assay, such as sample collection, shipping or processing procedures, genetic factors or dietary factors. Threats to assay validity or patient acquisition strategies might be evaluated. Because the number of samples is limited, it is tempting to describe pre-validation studies as feasibility or pilot studies to achieve one or more of these non-pivotal objectives, but this strategy increases the total number of samples used before critical decisions can be made.

Pivotal goals in early validation trials include: establish the feasibility of the use of a marker as a classifier (question: can a useful classifier be constructed, which may not be obvious with a high-dimensional marker such as SELDI); assess the adequacy of sensitivity and specificity in patients (question: are the sensitivity and specificity good enough for development as a population screening tool); assess the adequacy of sensitivity and specificity in tissue (question: does the marker in the sampled medium represent the marker in pre-cancerous or cancerous tissue). All of these pivotal goals suggest either choices about how the marker will be used or if it warrants further development. Non-pivotal goals can be included as secondary objectives.

Some trial designers contend that all objectives of a trial requiring the assessment of sensitivity and specificity can be accomplished by constructing the ROC curve, and that the area under the ROC curve (AUC), or a portion of it, constitutes an appropriate univariate outcome against which a trial may be powered. While the ROC curve is a useful tool for describing the attributes of some classifiers (those that are measured on a continuous scale), it is not particularly useful in population screening bio-marker validation trial design, because: the ROC curve does not naturally conform to the decision-making context of the biomarker development program; there is no standard for how big an AUC, or how big a change in the AUC, is *scientifically* significant; most of the ROC curve is irrelevant to a population screening marker.

To demonstrate this last point, consider the ROC curve presented in Figure 1, which corresponds to the serum biomarker example. Based on an initial, statistically significant t-test, this marker would generate sufficient scientific interest to move to an early validation study. The risk-minimizing decision rule is related to the ROC curve by Equation 2, demonstrating that, for a discriminator with this degree of separation, and under the specified prevalence estimates and cost assumptions, the sensitivity would have to be driven down to 0.75 before the specificity would be high enough to prevent an excess of false positive cases in a screening application. For most population screening applications, the specificity must be in excess of 0.95 to avoid a large number of false positives. Therefore, 19/20 of the ROC curve is not relevant to the decision.

There are several criticisms to the use of cost and prevalence data in the design of early validation trials:

### It is too early to consider these factors

If prevalence and cost are not considered, the decision to develop the marker as a population screen, clinical marker or panel component is simply delayed, and a trial designed to identify modest discrimination will have to be repeated with a larger sample size.

### There will never be a consensus on the estimate of C_−_/C_+_

It is not necessary that a particular value be chosen in advance and treated as inviolate. It is useful, however, to check if, given the prevalence estimate (about which there should be agreement), the proposed sensitivity and specificity criteria imply an assumption about the C_−_/C_+_ ratio that is patently absurd. It should be stressed that these costs are always inherent in statistical classification, so even if they are not explicitly considered, they are implicitly present, interacting with the ratio (1 − π*)/*π in Equation 1. Given that human beings are not very good at mentally manipulating ratios involving very small or very large numbers, it is prudent to perform a reality check.

### It is only necessary to do better than an existing marker

Since many markers used in clinical applications have truly dismal characteristics, the utility of using scarce resources to replace a mediocre marker with a somewhat better but still mediocre marker is questionable. Realistic decisions are made on the basis of analyses of absolute benefits to clinical or epidemiological applications, independent of the scientific interest in the biology of the marker.

### All the information can be derived from the ROC curves

If a marker is inherently continuous, the ROC curve does provide a useful tool for investigating some its features. But, the visual qualities of the ROC curve tend to overemphasize the importance of the sensitivity in the region of lower specificity, where a practical marker is unlikely to lie. Hypothesis tests based on the area under the ROC curve have the same problem; the AUC is dominated by the the region of the ROC far to the right of a useful clinical or population screen. Hypothesis tests based on partial areas require a decision about which portion of the ROC curve is relevant, returning us to the original problem of asserting the most important value of specificity, which is systematically done by calculating the optimal value of ROC’ using cost and prevalence assumptions.

### The cost criteria will tend to set the bar too high

One of the early decision points of a marker is population screen versus clinical tool versus panel component. The bar must be set high to use a marker as a population screen, because this is the most rigorous application. Many clinicians would argue, for instance, that the prostate specific antigen, which currently represents the gold standard for prostate cancer screening, is insufficiently specific for that application. Deciding a marker is not useful as a standalone population screen does not mean it should not be developed. But, the research programs for population screens, clinical tools and panel components are quite different, and the decision about which course should be followed should be made as soon as possible in the development program.

In this paper, we have asserted that, given the developmental context and high cost of samples, even early validation trials on markers should be designed to satisfy decision-theoretic criteria. While some reliance on heuristics is probably unavoidable, we have also suggested that, while many of the issues in trial design are resistant to analytic solutions, relatively simple Monte Carlo simulations can be be used to estimate the operating characteristics of trials, even those where some model-building is inherent. We believe that, if early trials are considered as part of a development process and are more rigorously designed using the tools we have presented, that the ultimate cost of design programs can be reduced, bringing potentially valuable markers to the clinic and the general community sooner.

## Figures and Tables

**Table 1:** Results of a test that classifies cases versus controls. The numbers in parentheses are 95% likelihood ratio confidence intervals for proportions.

	To Control	To Case
From Control	19/20 (0.75,0.99)	1/20
From Case	5/20	15/20 (0.51,0.91)
